# Supplementary material for: Maximal Efficacy of Alternative Splicing is Ensured by Balanced Efficiency of U1 and U2AF
Source: bioRxiv. 2025 Apr 30:2025.04.24.650448. Preprint. [Version 2] doi: 10.1101/2025.04.24.650448 (PMC12478324; doi:10.1101/2025.04.24.650448)
Supplement: 1 [file NIHPP2025.04.24.650448V2-supplement-1.pdf]

bioRxiv preprint doi: https://doi.org/10.1101/2025.04.24.650448; this version posted April 30, 2025. The copyright holder for this preprint (which was not certified by peer review) is the author/funder. This article is a US Government work. It is not subject to copyright under 17 USC 105 and is also made available for use under a CC0 license.

Supplemental Information

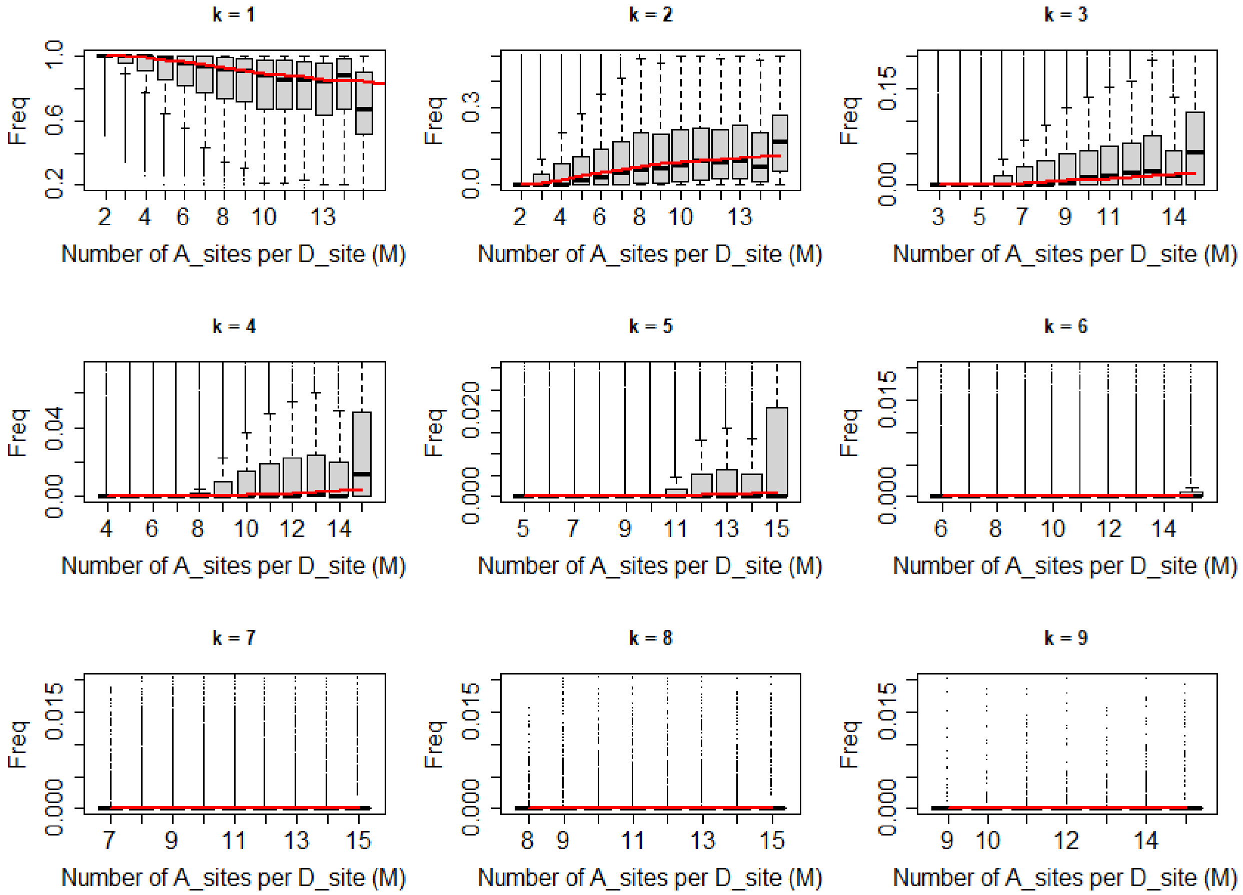

Fig S1. Boxplots of the distribution of frequency of alternative acceptor sites with fixed k and increasing M from RNA-seq data. k is the rank of alternative acceptor sites. M is total number of alternative acceptor sites. Red curve is median frequency calculated from $W(0.14)$.

bioRxiv preprint doi: https://doi.org/10.1101/2025.04.24.650448; this version posted April 30, 2025. The copyright holder for this preprint (which was not certified by peer review) is the author/funder. This article is a US Government work. It is not subject to copyright under 17 USC 105 and is also made available for use under a CC0 license.

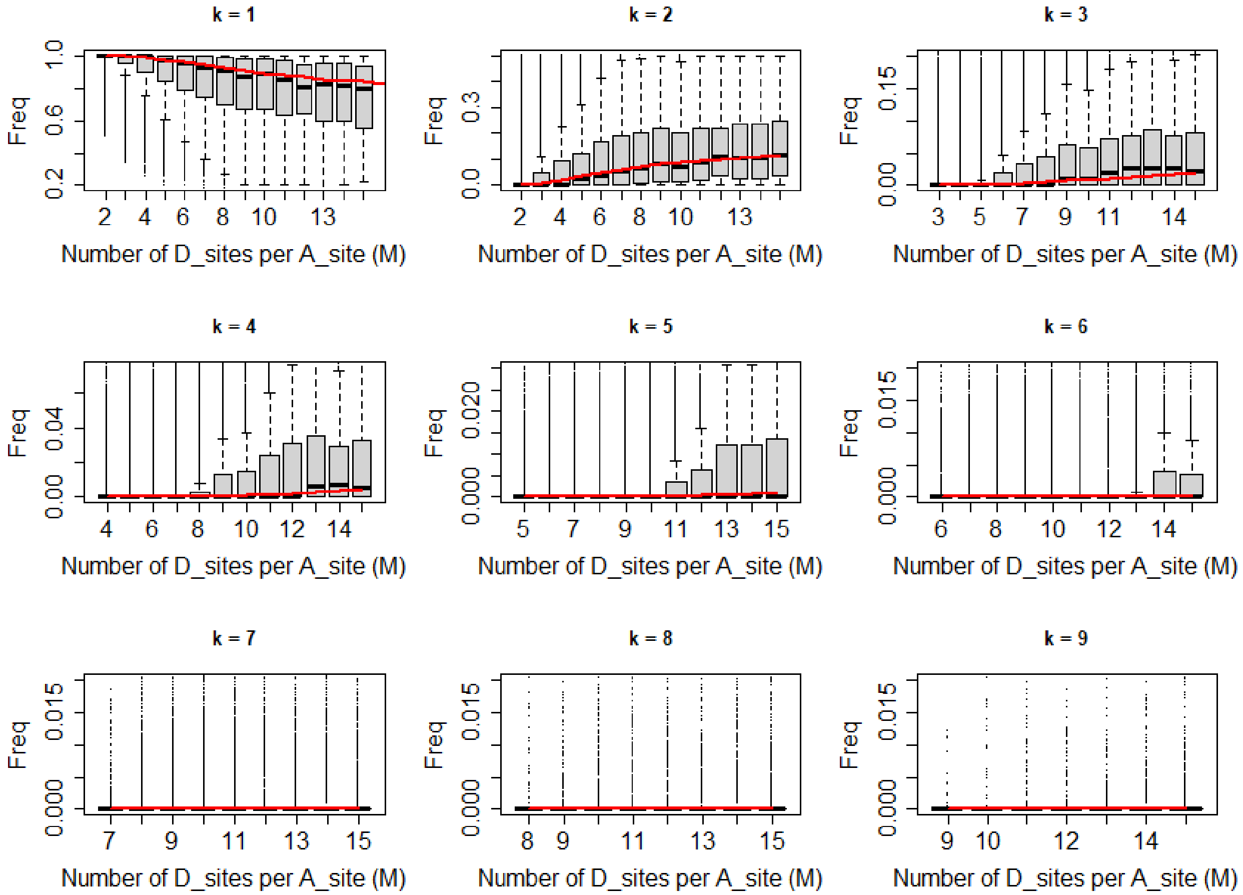

Fig S2. Boxplots of the distribution of frequency of alternative donor sites with fixed *k* and increasing *M* from RNA-seq data. *k* is the rank of alternative donor sites. *M* is total number of alternative donor sites. Red curve is median frequency calculated from *W*(0.14).

bioRxiv preprint doi: https://doi.org/10.1101/2025.04.24.650448; this version posted April 30, 2025. The copyright holder for this preprint (which was not certified by peer review) is the author/funder. This article is a US Government work. It is not subject to copyright under 17 USC 105 and is also made available for use under a CC0 license.

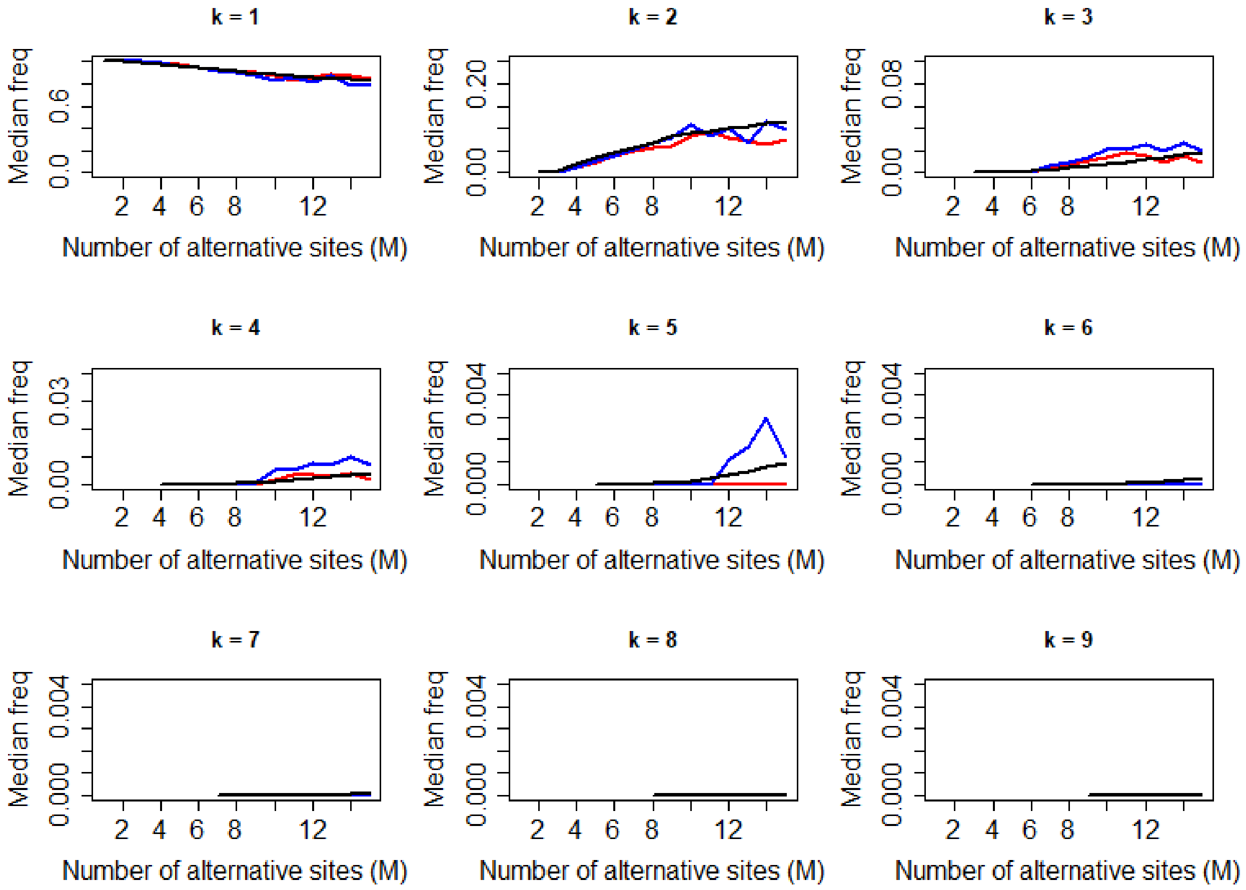

Fig S3. The distribution of median frequency of alternative acceptor and donor sites with fixed *k* and increasing *M* from colorectal cancer RNA-seq data (GSE50760). *k* is the rank of alternative splice sites. *M* is total number of alternative splice sites. Red line represents alternative acceptor sites. Blue line represents alternative donor sites. Black line represents simulated distribution from *W*(0.14).

bioRxiv preprint doi: https://doi.org/10.1101/2025.04.24.650448; this version posted April 30, 2025. The copyright holder for this preprint (which was not certified by peer review) is the author/funder. This article is a US Government work. It is not subject to copyright under 17 USC 105 and is also made available for use under a CC0 license.

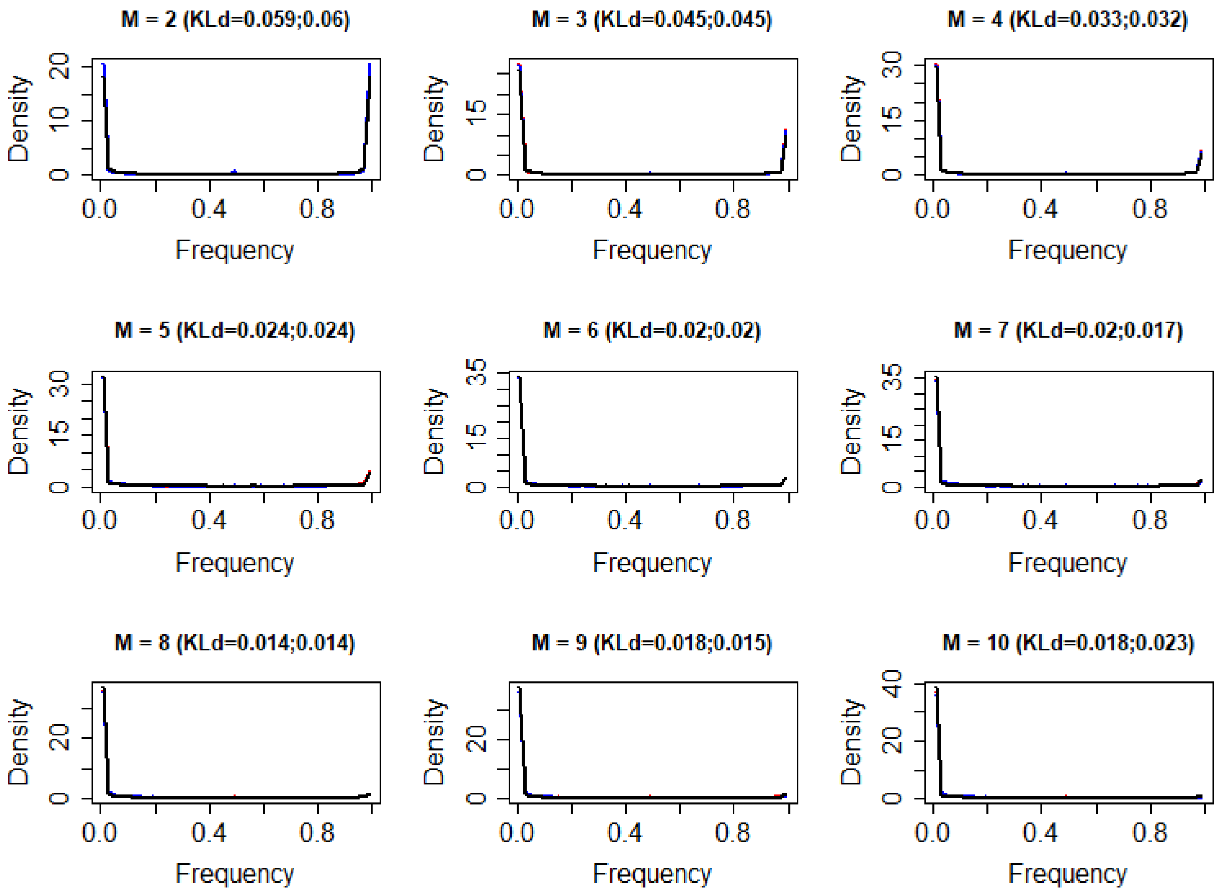

Fig S4. The frequency distribution of all alterative splice sites from colorectal cancer RNA-seq data (GSE50760) and simulated data for M=2:10. M is the number of alternative donor and acceptor sites. Red curves represent alternative acceptor sites, blue curves represent alternative donor sites, black curves represent simulated data from *W*(0.14). KLd is the Kullback-Leibler divergence between the two distributions. The first number in parentheses is KLd between the frequency distribution of alternative acceptor sites and simulated data from *W*(0.14). The second number in parentheses is KLd between the frequency distribution of alternative donor sites and simulated data from *W*(0.14). The three curves almost exactly overlap.

bioRxiv preprint doi: https://doi.org/10.1101/2025.04.24.650448; this version posted April 30, 2025. The copyright holder for this preprint (which was not certified by peer review) is the author/funder. This article is a US Government work. It is not subject to copyright under 17 USC 105 and is also made available for use under a CC0 license.

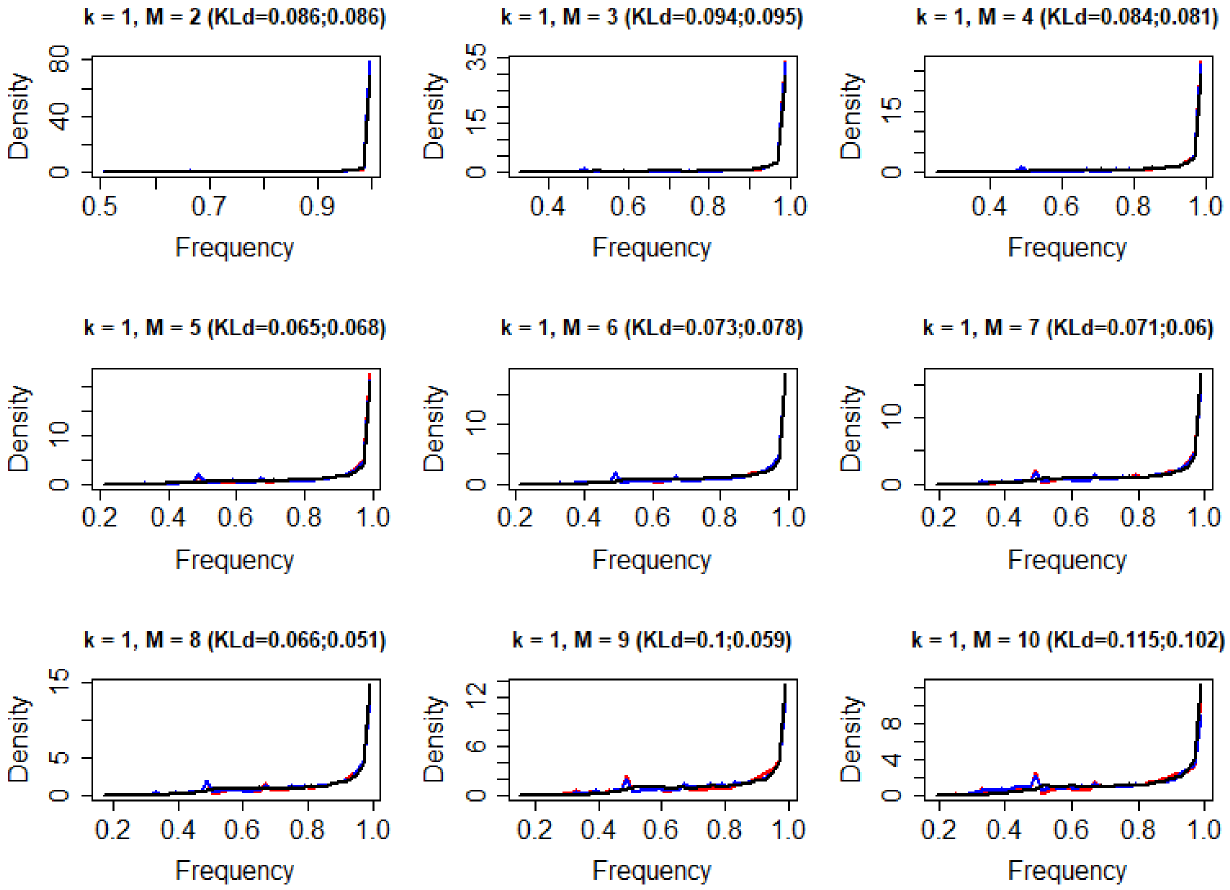

Fig S5. The frequency distribution of the most dominant alterative splice sites (k=1). M is the number of alternative donor and acceptor sites. The experimental data is from colorectal cancer RNA-seq data (GSE50760). Red curves represent alternative acceptor sites, blue curves represent alternative donor sites. Black curves represent simulated data from $W(0.14)$. KLd is the Kullback-Leibler divergence between two distributions. The first number in parentheses is the KLd between the frequency distribution of alternative acceptor sites and simulated data from $W(0.14)$. The second number in parentheses is KLd between the frequency distribution of alternative donor sites and simulated data from $W(0.14)$. The three curves almost exactly overlap.

bioRxiv preprint doi: https://doi.org/10.1101/2025.04.24.650448; this version posted April 30, 2025. The copyright holder for this preprint (which was not certified by peer review) is the author/funder. This article is a US Government work. It is not subject to copyright under 17 USC 105 and is also made available for use under a CC0 license.

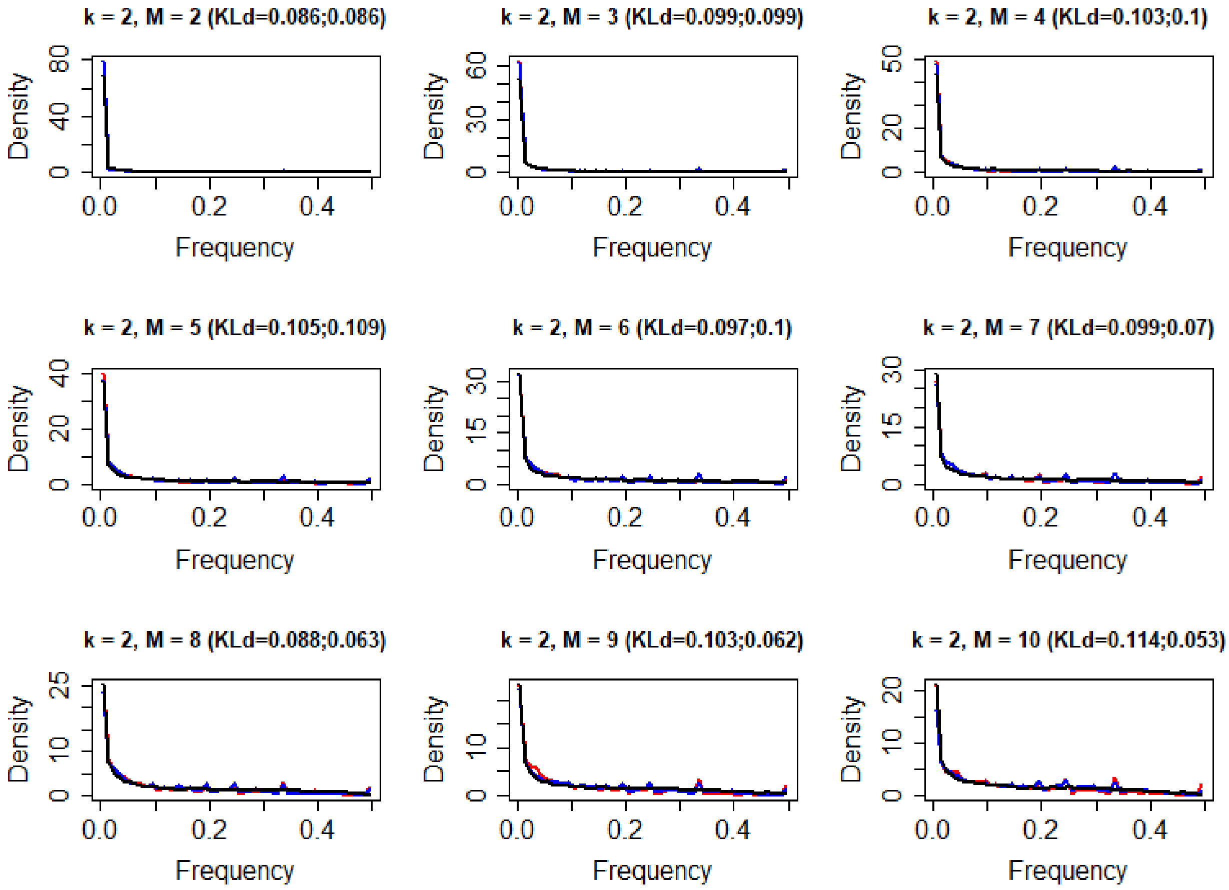

Fig S6. The frequency distribution of the second most dominant alterative splice sites (k=2). M is the number of alternative donor and acceptor sites. The experimental data is from colorectal cancer RNA-seq data (GSE50760). Red curves represent alternative acceptor sites, blue curves represent alternative donor sites. Black curves represent simulated data from *W*(0.14). KLd is the Kullback-Leibler divergence between two distributions. The first number in parentheses is the KLd between the frequency distribution of alternative acceptor sites and simulated data from *W*(0.14). The second number in parentheses is KLd between the frequency distribution of alternative donor sites and simulated data from *W*(0.14). The three curves almost exactly overlap.

bioRxiv preprint doi: https://doi.org/10.1101/2025.04.24.650448; this version posted April 30, 2025. The copyright holder for this preprint (which was not certified by peer review) is the author/funder. This article is a US Government work. It is not subject to copyright under 17 USC 105 and is also made available for use under a CC0 license.

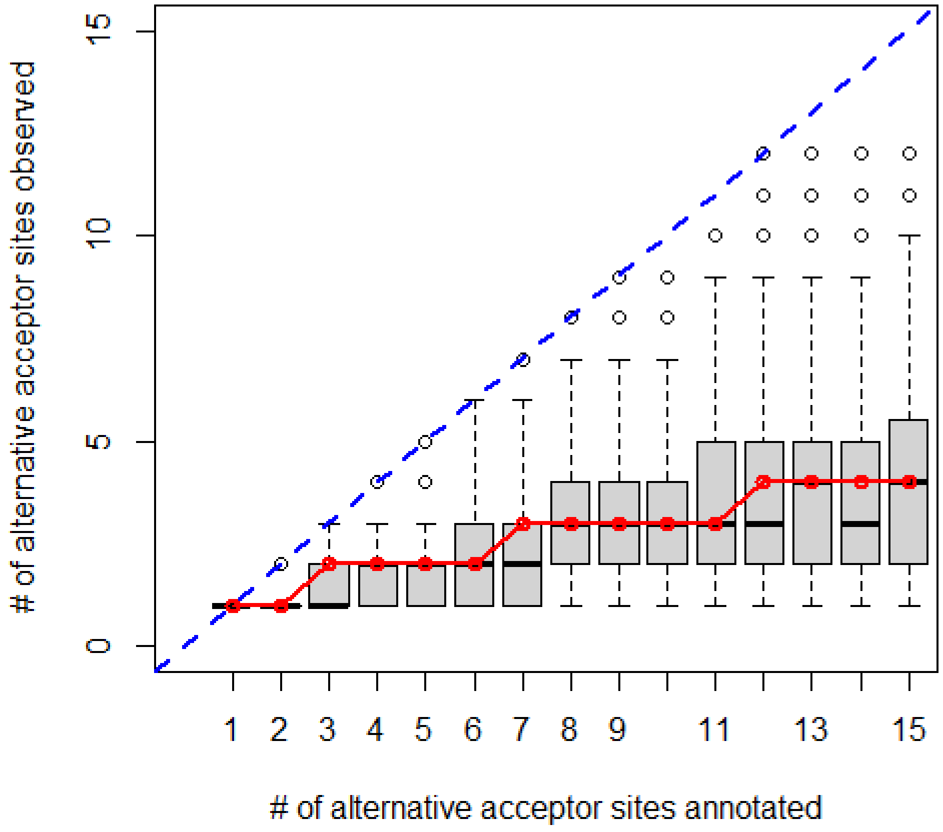

Fig S7. Boxplots of the distribution of the number of alternative acceptor sites observed in one sample versus those annotated. The boxplot is the observed result from our RNA-seq data. The red curve is the expected median calculated from $W(0.14)$.

bioRxiv preprint doi: https://doi.org/10.1101/2025.04.24.650448; this version posted April 30, 2025. The copyright holder for this preprint (which was not certified by peer review) is the author/funder. This article is a US Government work. It is not subject to copyright under 17 USC 105 and is also made available for use under a CC0 license.

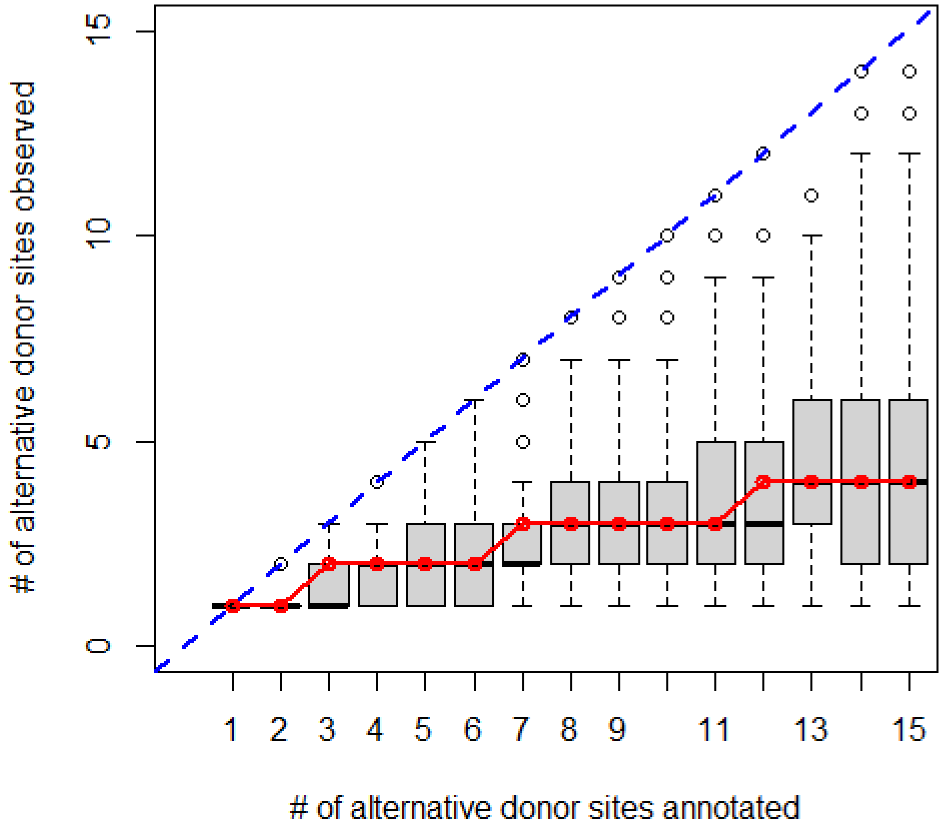

Fig S8. Boxplots of the distribution of the number of alternative donor sites observed in one sample versus those annotated. The boxplot is the observed result from our RNA-seq data. The red curve is the expected median calculated from $W(0.14)$.
